# Association between rs12045440 Polymorphism in the CAPZB Intron and Serum TSH Concentrations in Chinese Thyroid Tumor Patients

**DOI:** 10.1155/2015/250542

**Published:** 2015-07-26

**Authors:** Shouhao Feng, Shengli Lin, Jidong Zou, Yulong Wang, Qinghai Ji, Zhenghua Lv

**Affiliations:** ^1^Department of Otorhinolaryngology-Head and Neck Surgery, Provincial Hospital Affiliated to Shandong University, Jinan 250021, China; ^2^Department of Head and Neck Surgery, Cancer Hospital, Fudan University, Shanghai 200032, China; ^3^Department of Oncology, Shanghai Medical College, Fudan University, Shanghai 200032, China; ^4^Department of General Surgery, Zhongshan Hospital, Fudan University, Shanghai 200032, China

## Abstract

The aim of this study was to investigate the possible influence of different genotypes of the lead single nucleotide polymorphisms (SNPs) rs10917468 and rs12045440 in the CAPZB gene on the thyroid function in papillary thyroid carcinoma (PTC) and benign thyroid neoplasm (BN) patients. In the study, a significant association was detected between rs12045440 and serum TSH concentrations in thyroid tumor patients (*p* = 0.001). After the adjustment of relevant covariates, the difference between the mean serum TSH levels in different genotypes of rs12045440 was still significant in the BN group (*p* = 0.003) but was not significant in the PTC cases (*p* = 0.115). No significant association of rs10917468 with TSH levels was found. The SNP rs12045440 was associated with the serum TSH concentrations in Chinese thyroid tumor patients, especially in benign thyroid tumor cases.

## 1. Introduction

A thyroid nodule, the most common thyroid disease, can be a hyperplastic benign tumor (BN) or a malignant lesion. In some studies, thyroid nodules were discovered by sonography or autopsy in approximately 20% to 50% of the population, and the ratio of females to males ranged from 5 : 1 to 10 : 1 [1–4]. Although the proportion of the malignant thyroid nodules is low (age-adjusted global incidence rates range from 0.5 to 10 cases per 100,000 persons), thyroid carcinoma is the fifth leading cancer in females and the most common endocrine malignancy [5, 6]. Papillary thyroid carcinoma (PTC), accounting for 85–90% of all thyroid cancers, is the most common type of thyroid malignancy [7]. Both nodular goiters and PTCs are derived from thyroid follicular epithelial cells that are responsible for thyroid hormone synthesis. It has been postulated that both nodular goiters and PTCs are complex diseases, and that the development of thyroid nodules involves complex interactions between environmental and genetic factors. Significantly different concordance rates for simple goiter in female monozygotic and dizygotic twins in epidemiological studies strongly suggest a genetic component in the etiology of simple goiter [8–10]. Familial aggregation of thyroid carcinoma indicates an involvement of a genetic component in the tumorigenesis [11, 12]. The associations of familial thyroid cancers with a number of rare Mendelian cancer syndromes provide direct evidence for a genetic predisposition [13]. However, the etiologies of BN and that of PTC are still unknown.

Thyrotropin (thyroid-stimulating hormone, TSH), which binds specifically to the TSH receptor, plays a major role in the regulation of thyroid cell growth and synthesis and secretion of thyroxine (T4) and triiodothyronine (T3). Some researchers found that the risk of malignancy in a thyroid nodule increases with the serum TSH concentrations [14–16]. Although there is little convincing evidence that TSH contributes to the initiation of thyroid carcinogenesis, TSH suppression by LT4 is recommended in the treatment of differentiated thyroid cancer (DTC) to reduce the risk of the recurrence of and death from thyroid cancer [17].

Under the control of the hypothalamic-pituitary axis, the thyroid hormones regulate the concentration of TSH via negative feedback. In healthy subjects, the individual reference ranges for TSH and free T4/T3 are very narrow, with significant interindividual differences [18].

A genome-wide association study (GWAS) in Germany reported 4 genetic loci associated with thyroid volume and goiter risk [19]. Intriguingly, two independent loci associated with goiter risk were found within the CAPZB region on chromosome 1p36: the lead SNP rs10917468 at the locus upstream of CAPZB and the lead SNP rs12045440 located in the first of the 9 introns of CAPZB.

CAPZB, which is also known as capping protein (actin filament) muscle Z-line subunit *β*, encodes the *β* subunit of the barbed-end actin binding protein. The capping protein (CP) is a heterodimer composed of an *α* and *β* subunit. CAPZ*β* can exist as 3 unique isoforms arising from distinct splicing mechanisms. The *β*1 subunit is the predominant isoform of muscle tissues, with 31.4 kDa and 277 amino acids in length, while the *β*2 isoform is predominant in nonmuscle tissues, with 30.6 kDa and 272 amino acids in length. The *β*3 subunit is 301 amino acids in length, showing an N-terminal extension of 29 amino acids relative to the *β*2 isoform. Its mRNA is detected in testis, as a component of the cytoskeletal calyx of the mammalian sperm head [20–22]. CP functions to cap actin filaments at barbed-ends and decreases the polymerization of G-actin to F-actin and thus inhibits the actin filament elongation. It is essential for actin-based motility in filopodia as well as in lamellipodia, both of which are important for locomotion in many types of migrating cells [23–25].

It is speculated that the causative sequences in the linkage equilibrium of the lead SNPs influence the activity of the gene promoter or cause an amino acid exchange. As a consequence, the altered uncapping activity of the capping protein results in a reduced or accelerated thyroglobulin engulfment by filopodia and, in turn, the change of concentrations of thyroid hormone and TSH. This leads to a modification of the thyrocyte proliferation and an increased or decreased goiter risk. It has been postulated that the CAPZB gene is involved in the risk of goiter. However, there are no further studies to validate the postulation, and whether the CAPZB gene plays a role in the tumorigenesis still remains unknown.

The aim of our study was to test the associations between the lead SNPs rs10917468 and rs12045440 (the genetic information of the SNPs was available at the website: http://genome.ucsc.edu/) and the risk of PTC and BN and to investigate the possible influence of different genotypes on the thyroid function in PTC and BN patients.

## 2. Materials and Methods

### 2.1. Study Population

In this case-control study, the case population consisted of 870 PTC and 513 BN patients who underwent surgeries at the Department of Head and Neck Surgery, Cancer Hospital, Fudan University, Shanghai, China, from January 2010 to December 2010. All the case subjects were ethnically Chinese Han, living in Shanghai, Jiangsu, Zhejiang, and surrounding regions, Eastern China. The enrollment criteria were as follows: histologically identified diagnosis by the Department of Pathology of the Cancer Hospital of Fudan University, no previous surgical or medical treatment of a thyroid disease, no history of familial thyroid cancer, and no radiation exposure. The control population consisted of 1244 cancer-free healthy subjects recruited from the Taizhou Longitudinal Study in the same period, who fulfilled the enrollment criteria, including the criterion of having no history of cancer or thyroid disease [26]. The demographic data (including age, gender, and ethnicity) and environmental exposure history (including radiation, smoking, and alcohol consumption) of each enrolled subject were collected via personal interview.

In the Cancer Hospital of Fudan University, all the patients with thyroid tumors received an ultrasound examination and a preoperative serum thyroid function measurement with the chemiluminescence immunoassay, including TSH, free T4 (FT4), free T3 (FT3), thyroglobulin (TG), thyroglobulin antibodies (TGAb), and thyroid peroxidase antibodies (TPOAb). Fine needle aspiration biopsy and Computed Tomography were not performed routinely. During the operation, pathological frozen section examination was performed routinely after lobectomy. When the frozen section examination reported a malignant diagnosis, an ipsilateral level VI lymph node dissection was performed. If malignant lesions were diagnosed in both lobes of the thyroid by frozen section examination, a total thyroidectomy and a bilateral level VI lymph node dissection were performed. If a benign or undetermined nodule was found in the contralateral lobe by ultrasound examination, a subtotal lobectomy with frozen section was also performed during the operation. We described the management of thyroid tumors in our hospital in our previous publications [27, 28].

### 2.2. Ethics Statement

The Ethical Committee of the Cancer Hospital of Fudan University approved the study protocol, and all patients were provided written informed consent.

### 2.3. DNA Extraction and Genotyping

Genomic DNA was extracted from peripheral blood samples using the Qiagen Blood Kit (Qiagen, Chatsworth, CA, USA) according the instructions of manufacturer. Extracted DNA was dissolved in 100 uL Tirs-HCl buffer (10 mmol/L, pH8.0) containing 1 mmol/L EDTA and was stored at −20°C. We measured the DNA concentrations with the help of NanoDrop 1000 spectrophotometer (Thermo Scientific) and diluted the DNA samples to about 10 ng/*μ*L before using them.

Genotyping was performed with the SNaPshot Multiplex System (Applied Biosystems, California, USA). Primers for the PCR amplification were designed with the software Primer Premier 5.0 according to the reference sequence available from the dbSNP database (http://www.ncbi.nlm.nih.gov/SNP/). The genotyping results of all the SNPs were obtained with an ABI PRISM 3730 Genetic Analyser and Peak Scanner software v1.0 (Applied Biosystems, California, USA). In each 96-well plate, we randomly selected 4 DNA samples as internal positive controls. To maintain quality control, three experimenters, unaware of the study group of each sample, read the genotyping results independently for quality control. If the peak which represented the SNP was too low to distinguish, we repeated the genotyping of the sample. No inconsistent or abnormal result was found in the positive controls, and all the samples of tumors and control groups were genotyped successfully.

### 2.4. Statistical Analysis

All the statistical analyses were performed with the SPSS Software version 12.0 (SPSS, Chicago, IL, USA). Differences in selected variables and Hardy-Weinberg equilibrium were evaluated with the Chi-square test or Wilcoxon rank-sum test as appropriate. The odds ratios (ORs) and 95% confidence intervals (CIs) were calculated using the binary logistic regression analysis to determine associations between SNP genotypes and the risk of PTC and BN. A linear logistic regression model was used to assess the associations between the SNP genotypes and the TSH concentrations adjusted to gender, age, FT3, FT4, TG, TGAb positivity, TPOAb positivity, and, where appropriate, pathologic type. Serum concentrations of TSH, FT3, FT4, and TG were log-transformed, and the TPOAb/TGAb statuses were dichotomized according to the cut-off value provided by the assay manufacturer prior to the analysis. A two-tailed *p* value of less than 0.05 was considered statistically significant. The genotyped SNPs were in the Hardy-Weinberg equilibrium in the PTC, BN, and control groups (*p* > 0.05).

## 3. Results 

### 3.1. Clinical Characteristics of the Study Population

As shown in Table 1, the final analysis included 870 PTC patients, 513 BN patients, and 1244 healthy controls (the tumor group consisted of PTC and BN cases). Among the 513 BN cases, there were 320 nodular goiter (62.4%), 101 follicular adenoma (19.7%), and 92 cases with both nodular goiter and follicular adenoma (17.9%). The PTC cases were younger than the BN cases (mean ± SD 45.73 ± 11.63 years versus 48.25 ± 12.05 years, *p* < 0.001), while the mean age of the control group had the highest value (mean ± SD 51.96 ± 13.90 years). The female to male ratio was lower in the control group than in PTC and BN groups (*p* < 0.001), while there were no significant differences between PTC and BN cases regarding gender distribution (*p* = 0.439).

### 3.2. Associations between Candidate SNPs and Risk of Thyroid Tumors

Table 1 showed that distributions of age and gender in PTC, BN, and control groups were significantly different. To adjust the effects of the different distributions of age and gender, we analyzed the associations between the candidate SNPs within the CAPZB gene and the risk of PTC/BN in a binary logistic regression model. However, as shown in Table 2, no significant differences were found.

### 3.3. The Characteristics of Preoperative Thyroid Function Measurement in Thyroid Tumor Patients

All the patients with thyroid tumors received a preoperative serum thyroid function measurement with the chemiluminescence immunoassay.  Table 3 presents the characteristics of the thyroid function measurements. The PTC cases had higher mean serum TSH levels than the BN cases (2.14 ± 1.68 mIU/L versus 1.79 ± 2.07 mIU/L, *p* < 0.001). The TGAb and TPOAb statuses were dichotomized according to the cut-off value provided by the assay manufacturer; as a result 12.2% and 10.26% cases had high TGAb and TPOAb values, respectively. Due to incomplete data, the TGAb statuses of one BN patient and four PTC patients as well as the TPOAb statuses of three BN and six PTC patients were unknown. Of the 1383 tumor cases, the PTC patients had higher rates of positive TGAb and positive TPOAb than the BN patients (15.70% versus 6.25%, *p* < 0.001; 12.04% versus 7.25%, *p* = 0.005).

### 3.4. Association between rs12045440 Polymorphism and the Serum TSH Levels in PTC and BN Groups

Table 4 summarizes the associations between rs12045440 polymorphism and the serum TSH levels in tumor patients; it shows that the rs12045440 polymorphism substantially affected the results in the BN group (*p* = 0.003) but not in the PTC group (*p* = 0.115). No significant association between rs10917468 and the TSH levels was found in the PTC and the BN groups. The associations between the SNPs and the serum FT3/FT4 levels were also calculated, but no statistically significant results were obtained.

## 4. Discussion

The SNP rs12045440 is located within the first of the nine introns of CAPZB, and the minor allele G was identified to be associated with decreased thyroid volume and reduced goiter risk in the European population in a GWAS by Teumer et al. [19]. Our study showed no significant differences in distribution of rs12045440 genotypes in 1244 healthy subjects, 513 benign thyroid tumor cases, and 870 PTC patients (see Table 2). However, the mean concentration of the wild-type TT genotype of rs12045440 was 0.34 mIU/L lower than that of the alternative homozygote GG genotype in thyroid tumor patients, suggesting a significant association of SNP rs12045440 with the serum TSH concentration after the adjustment to the relevant covariates (mean ± SD. 1.91 ± 1.62 mIU/L versus 2.25 ± 1.65 mIU/L, *p* = 0.001) (see Table 4). In the BN group, the difference between the wild-type TT genotype and the alternative homozygote GG genotype regarding the mean TSH concentration was 0.43 mIU/L (mean ± SD. 1.57 ± 1.13 mIU/L versus 2.00 ± 1.53 mIU/L, *p* = 0.003) (see Table 4); this difference was not significant in the PTC group (mean ± SD. 2.10 ± 1.83 mIU/L versus 2.38 ± 1.7 mIU/L, *p* = 0.115) (see Table 4). Nevertheless, the GWAS carried out in Germany did not reveal an association between the SNP rs12045440 and serum TSH concentration.

In thyroid nodular diseases, a higher TSH concentration was seen as a risk factor for malignancy, and our study confirmed this belief. Some researchers speculated that the higher TSH concentration may contribute to the carcinogenesis. If the hypothesis was true, the distribution of the GG genotype of rs12045440 should be wider in PTC cases than in BN subjects. However, our analysis showed no differences regarding the distribution of rs12045440 genotypes in PTC and BN cases, whereas the GG genotype of rs12045440 was associated with a higher TSH concentration. The mechanism of carcinogenesis is complex and we cannot arbitrarily reject the above-mentioned hypothesis merely on the basis of our research. Hence, we prefer an alternative hypothesis; we hypothesize that the malignant transformation of thyroid follicular epithelium cells occurs, resulting in various physiological, biochemical, and metabolic changes, including an increase in the TSH concentration. The effect of the GG genotype of rs12045440 on the TSH concentration might be concealed by or interfere with some unknown changed factors which may cause malignancy. Despite the significant association between the SNP rs12045440 and the TSH concentration in thyroid tumor patients, we did not make thyroid function measurements in healthy subjects, and the association between rs12045440 and the TSH concentration in healthy subjects cannot be determined.

Teumer et al. inferred that SNP rs12045440 was in linkage disequilibrium of at least one yet-unidentified nonsynonymous SNP, causing an amino acid exchange in a *β* subunit. However, sequence polymorphisms in introns can regulate the expression of a gene in other ways, independently of exon sequence changes. The minor allele of rs12045440 may downregulate the expression of *β* subunit in thyrocytes, which results in enhanced uncapping activity. As a consequence, thyroglobulin engulfment by filopodia increases, leading to increased T3/T4 release. Subsequently, the thyroid volume is reduced and the goiter risk is lowered. When the volume of follicles becomes too small, the T3/T4 levels sink below the lower limits of the physiologically normal range, and the TSH concentration is increased under the control of the hypothalamic-pituitary axis. Experimental studies are needed to investigate the role of rs12045440 in the regulation of the TSH concentration. In the new equilibrium, the concentrations of FT3 and FT4 are inconspicuous. Consistent with the results of the GWAS, our analysis of the data demonstrated no associations between the FT3/FT4 concentrations and the different genotype groups.

Teumer et al. also identified another independent genetic locus which was associated with the thyroid volume and goiter risk, as their lead SNPs were not in significant linkage disequilibrium (*r* ^2^ = 0.004). Located upstream of CAPZB, rs10917468 represented the lead SNP for goiter, whereas rs12138950 was the lead SNP for thyroid volume. The two lead SNPs were in strong linkage disequilibrium with the SNP rs10917469 (rs10917468: *r* ^2^ = 0.60; rs12138950: *r* ^2^ = 1.00, resp.); the minor allele G of the two lead SNPs was strongly associated with decreased serum TSH concentration in a GWAS of Caucasian 7 population by Panicker et al. [29]. It seems that the three SNPs belong to one common haplotype, and consequently the SNPs rs12138950 and rs10917468 were supposed to have an impact on the serum TSH level. We genotyped rs10917468 in our study, but the data did not reveal a significant association between the rs10917468 and the serum TSH concentration in PTC and BN cases. The existence or absence of a tumor and ethnic differences might be the sources of the inconsistencies. The investigated subjects of our study were ethnic Chinese Han with PTC or BN diseases, while those of the two above-mentioned GWASs were disease-free Caucasian. In addition, the rs10917468 may not completely represent the haplotype. Further investigations are required to identify the association between the genetic locus and the TSH concentration both in healthy subjects and in thyroid tumor patients.

Subclinical hypothyroidism, characterized by an elevated TSH level combined with normal references of thyroid hormones, is a strong indicator of risk for dyslipidemia, cardiovascular diseases, myocardial infarction, and atherosclerosis [30–32]. And a higher TSH concentration was a risk factor for malignancy in thyroid nodules, as is referred to above. To explore the susceptibility loci and genes related to TSH concentrations may help us better understand the pathogeneses of these disorders. However, little is known about the genes that contribute to the interindividual variations of the TSH concentration at present. Genome-wide association studies have identified five TSH susceptibility genetic loci: 8q12.1, 5q13.3, 1p36.13, 16q23, and 4q31 [19, 29, 33–35]. The lead SNPs of these loci were located within XKR4, within PDE8B, upstream of CAPZB, in a former “gee desert” that was recently demonstrated to encode a functional gene (LOC440389), and upstream of NR3C2. At the locus 1p36.13, one of the two independent blocks associated with thyroid volume and goiter risk that was reported to be located upstream of CAPZB, of which the lead SNPs Rs10917468 and Rs12138950 are in moderate LD with Rs10917477 (*r* ^2^ = 0.26 and 0.15, resp.), the lead SNP for TSH levels in Rawal and Teumer's meta-analysis. Yet our group confirmed the association between the second genetic locus, of which the lead SNP Rs12045440 is located in intron 1 of CAPZB, and the serum TSH concentrations in Chinese thyroid tumor patients. Our results support those of Teumer et al. and Panicker et al., indicating that the sequence polymorphisms in the CAPZB gene contribute to interindividual variations of the TSH concentration.

## 5. Conclusion

The SNP rs12045440 (1p36) within intron 1 of CAPZB was associated with serum TSH concentrations in Chinese thyroid tumor patients, especially in thyroid benign thyroid tumor cases. No significant association of the TSH concentrations with rs10917468 (1p36), located upstream of CAPZB, was found. The genotype distributions of rs12045440 and rs10917468 were not significant in PTC cases, BN patients, or healthy controls. Our case-control study contributed to a better understanding of the interindividual variations of the TSH concentration. To completely understand the role of CAPZB in the regulation of set-points of the pituitary-thyroid axis, further studies with larger sample sizes and functional studies are needed.

## Figures and Tables

**Table 1:** The age and gender distribution of the papillary thyroid cancer (PTC), benign thyroid tumor (BN), and control groups.

Characteristics	PTC	BN	Tumor	Control	*p* value	*p* value	*p* value	*p* value
	(*n* = 870)	(*n* = 513)	(*n* = 1383)	(*n* = 1244)	Tumor vs. Control	PTC vs. Control	BN vs. Control	PTC vs. BN
Age (mean ± SD)	45.73 ± 11.63	48.25 ± 12.05	46.66 ± 11.84	51.96 ± 13.90	<0.001^a^	<0.001^a^	<0.001^a^	<0.001^a^
Gender								
Male	237 (27.2%)	130 (25.3%)	367 (26.5%)	549 (44.1%)	<0.001^b^	<0.001^b^	<0.001^b^	0.439^b^
Female	633 (72.8%)	383 (74.7%)	1016 (73.5%)	695 (55.9%)				

^a^The control subjects were older than the BN cases (*p* < 0.001), and the BN cases were older than the PTC cases (*p* < 0.001).

^b^A similar gender distribution was found between PTC and BN (*p* = 0.439).

**Table 2:** Associations between the SNPs within the CAPZB gene and risk of thyroid tumors.

Candidate SNP	Sig.	Exp(*B*)	Sig.	Exp(*B*)	Sig.	Exp(*B*)	Sig.	Exp(*B*)
	Tumor vs. control	(95% CIs)	BN vs. Control	(95% CIs)	PTC vs. Control	(95% CIs)	BN vs. PTC	(95% CIs)
rs10917468	0.867	0.989 (0.873–1.122)	0.989	0.999 (0.845–1.181)	0.896	0.990 (0.858–1.143)	0.791	0.977 (0.823–1.160)

rs12045440	0.182	1.084 (0.963–1.219)	0.458	1.061 (0.907–1.242)	0.18	1.096 (0.959–1.252)	0.935	1.007 (0.857–1.182)

The values were calculated by analyzing the candidate age and gender adjusted SNPs in a logistic regression model.

**Table 3:** Preoperative serum thyroid function measurement statuses in PTC and BN groups.

Thyroid function measurement index	PTC (*n* = 870) Mean ± SD	BN (*n* = 513) Mean ± SD	Tumor (*n* = 1383) Mean ± SD	*p* value PTC vs. BN
FT3 (pmol/L)	4.37 ± 0.77	4.39 ± 0.70	4.38 ± 0.74	0.476
FT4 (pmol/L)	14.65 ± 2.32	14.59 ± 2.35	14.63 ± 2.33	0.967
TSH (mIU/L)	2.14 ± 1.68	1.79 ± 2.07	2.01 ± 1.84	<0.001
TG (ng/mL)	78.08 ± 174.20	166.47 ± 283.40	111.27 ± 225.21	<0.001
TGAb (IU/mL)	79.00 ± 277.86	32.56 ± 146.93	61.75 ± 238.78	<0.001
Positive TGAb (%)	136/866 (15.70%)^a^	32/512 (6.25%)^a^	168/1378 (12.2%)^a^	<0.001
TPOAb (IU/mL)	48.92 ± 162.61	29.40 ± 133.02	41.68 ± 152.54	<0.001
Positive TPOAb (%)	104/864 (12.04%)^a^	37/510 (7.25%)^a^	141/1374 (10.26%)^a^	0.005

FT3, free T3; fT4, free T4; TSH, thyrotropin; TG, thyroglobulin; TGAb, thyroglobulin antibodies; TPOAb, thyroid peroxidase antibodies.

^a^The statuses of TGAb in five cases and those of TPOAb in nine cases were not collected due to incomplete data.

**Table 4:** Association between rs1204540 and the serum TSH concentrations adjusted to relevant covariates.

	TSH concentration (mean ± SD. mIU/L)			Frequency (G)	Beta (SE.)	Sig.
	TT	TG	GG			
PTC	2.10 ± 1.83	2.10 ± 1.52	2.38 ± 1.71	0.351	0.056 (0.035)	0.115
BN	1.57 ± 1.13	1.94 ± 2.76	2.00 ± 1.53	0.348	0.152 (0.052)	0.003
Tumor	1.91 ± 1.62	2.04 ± 2.07	2.25 ± 1.65	0.35	0.097 (0.029)	0.001

Data were adjusted to gender, age, FT3, FT4, TG, and TGAb/TPOAb positivity.
